# Phloretin inhibits ferroptosis by restoring the antioxidant capacity of bovine adipose and muscle cells via the AMPK-PPAR signaling pathway

**DOI:** 10.1007/s44154-025-00263-4

**Published:** 2025-12-08

**Authors:** Jie Li, Enhui Jiang, Mengyang Zhang, Chuanying Pan, Chuzhao Lei, Lin Han, Xianyong Lan

**Affiliations:** 1 College of Animal Science and Technology, Northwest A and F University, Yangling, 712100 People’s Republic of China; 2 College of Food Science and Engineering, Northwest A and F University, Yangling, 712100 People’s Republic of China

**Keywords:** Phloretin, Ferroptosis, Bovine, Adipose and muscle cells, Transcriptome, Metagenomics

## Abstract

Ferroptosis has been increasingly implicated in adipose and muscle dysfunction, systemic metabolic disturbances, and several diseases in livestock, which necessitates effective and side-effect-free inhibition strategies. Phloretin, a dihydrochalcone with excellent antioxidant and anti-inflammatory properties, may have the potential to restrain cell ferroptosis. Herein, phloretin was verified to significantly inhibit (1S,3R)-RSL3-induced ferroptosis by reducing intracellular MDA, Fe^2^⁺, and ROS levels and restoring cell total antioxidant capacity in bovine and mouse preadipocytes or myoblasts. It also alleviated oxidative stress (OS), a vital inducer of ferroptosis, by restoring antioxidant enzyme activity in the above cells and obese mice. In vivo, phloretin gavage significantly reversed the trend where high-fat diet (HFD)-induced OS promoted the expression of ferroptosis-promoting genes and proteins (e.g., *ACSL4* and PTGS2) while inhibiting the expression of ferroptosis-negative regulators (e.g., *Fth1* and *Gpx4*). Unlike most flavonoids that exert anti-inflammatory or antioxidant activities by altering the gut microbiota composition, metagenomic sequencing analysis of cecal contents from phloretin-gavaged and HFD mice revealed that phloretin exerts its antioxidative and ferroptosis-inhibitory effects independent of modulating gut microbiota diversity. Further transcriptomic analyses of mouse adipose tissues revealed that phloretin alleviated ferroptosis in adipocytes by modulating the transcription of genes enriched in AMPK and PPAR signaling pathways, such as *Camkk2*. Hence, based on multi-omics analysis combined with in vivo and in vitro verification, phloretin effectively alleviated the OS to further inhibit ferroptosis of adipose or muscle cells through the AMPK-PPAR pathway, which can provide new research ideas for ameliorating adipose or myocyte dysfunction induced by ferroptosis in animals.

**Graphical Abstract:**

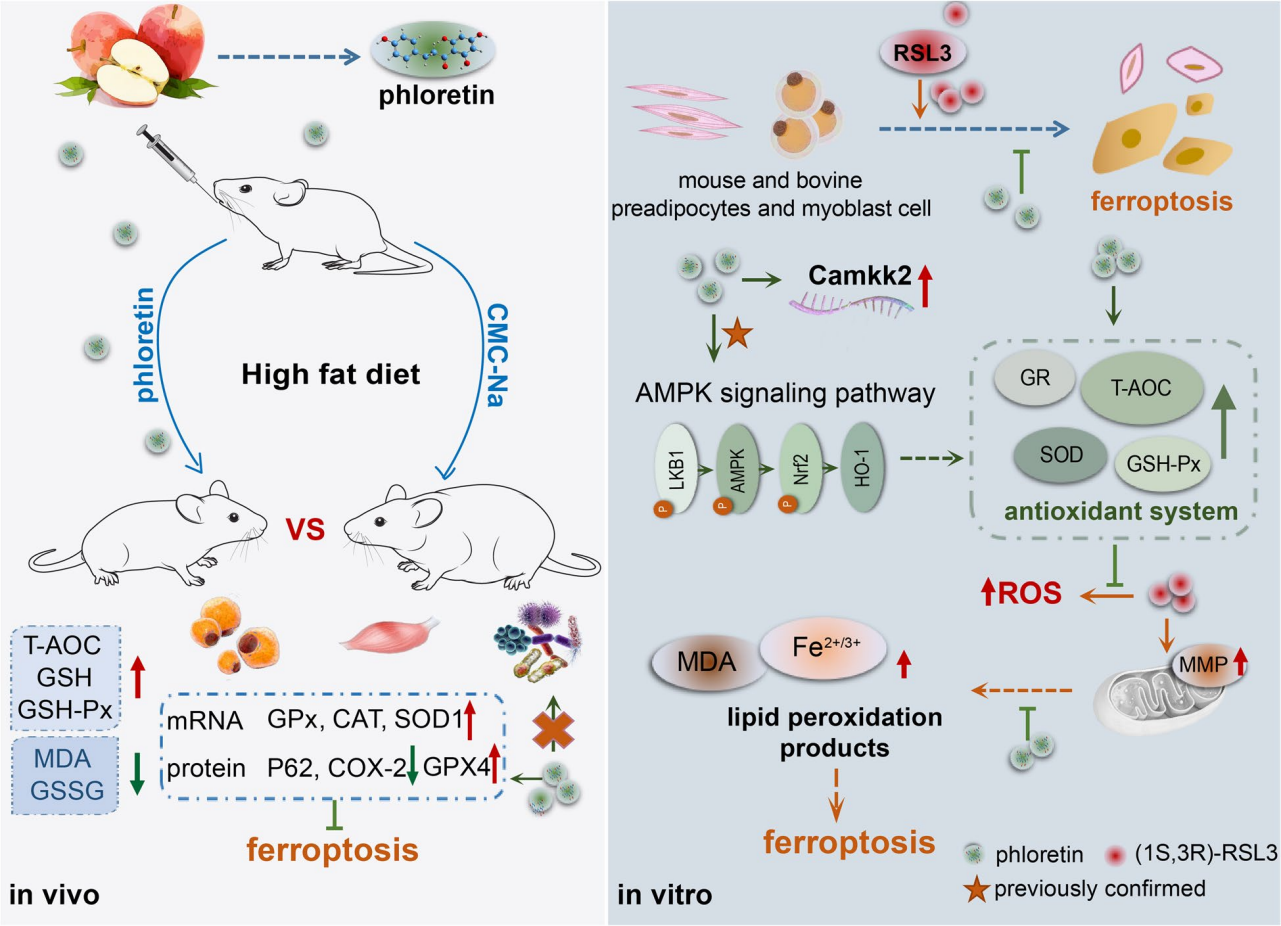

**Supplementary Information:**

The online version contains supplementary material available at 10.1007/s44154-025-00263-4.

## Introduction

Ferroptosis is a cell death driven by iron-dependent lipid peroxidation and oxidative stress (OS) (Dixon et al. 2012). As a cutting-edge research topic, ferroptosis is implicated in various pathological processes in livestock, such as the high-altitude hypoxia adaptation of yaks, oligospermia, endometrial hyperplasia and endometriosis (Gan et al. 2022; Zhang and Cui 2022). In animal husbandry, ferroptosis often occurs due to improper feeding practices, such as excess intake of iron and other micronutrients (Huo et al. 2022), as well as in response to thermal stress, inflammation, and OS in livestock (Cui et al. 2024). Additionally, exposure to heavy metals (e.g. chromium and cadmium), pesticide residues, and contamination with zearalenone can induce ferroptosis in animals (Gao et al. 2024; Wang et al. 2023; Zhong et al. 2024). Simultaneously, ferroptosis can destroy the survival and function of adipocytes and myocytes, the two primary cell types in livestock (Dawi et al. 2024; Fang et al. 2019; Li et al. 2023; Pope and Dixon 2023; Wang et al. 2022). Hence, nowadays, further exploration is needed to effectively inhibit ferroptosis and elucidate its molecular mechanisms in livestock.

Differ with apoptosis, necrosis, and autophagy, ferroptosis is stringently dependent on iron, reactive oxygen species (ROS), and the disruption of mitochondrial structure and function. The core molecular mechanism of ferroptosis is the imbalance between oxidative damage and antioxidant defense (Chen et al. 2023). Hence, the susceptibility to ferroptosis is contingent upon the intracellular antioxidant capacity and varies among different cell types (Mancardi et al. 2021). Cell death induced solely by ferroptosis can be reversed by using iron chelators, lipophilic antioxidants, and lipid peroxidation inhibitors (Lesjak et al. 2022). The triggers of ferroptosis are diverse, with currently identified induction mechanisms broadly classified into four categories, each with high specificity, including systemXc-inhibition, GPX4 inhibition/degradation/inactivation, depletion of reduced CoQ10 and excessive induction of lipid peroxidation by peroxides, PUFA, Fe^3+^, and Fe^2+^ (Stockwell 2022). Among these, oxidative stress is a significant trigger for the activation of ferroptosis and other ferroptosis-inducing pathways, such as Nrf2 and GPX4 pathway (Zheng and Conrad. 2020; Sabatier et al. 2023; Li et al. 2024). Although OS is not synonymous with ferroptosis (Stockwell 2022), the most common approach to inhibiting ferroptosis is currently the elimination of OS (Li et al. 2024; Liu et al. 2024).

Recently, natural bioactive compounds derived from plants have garnered significant attention due to their exceptional bioactivity and minimal toxicity. Phloretin, a small molecule dihydrochalcone compound (molecular weight: 274.27) abundant in apple (5 mg/100 g of fresh apple), has garnered significant attention due to its exceptional antioxidant and anti-inflammatory properties (Mariadoss et al. 2019; Comi et al. 2025). Its molecular characteristics underpin its potent antioxidant capabilities: 2,6-Dihydroxyacetophenone is the primary pharmacophore responsible for phloretin's potent antioxidant capabilities, enhanced by the carbonyl and -OH groups in ring A, whereas replacing an -OH group with a glycosyl group (as in phlorizin) diminishes this activity (Bentes et al. 2011). Phloretin exhibits pharmacological and nutritional potential in both medical and animal production contexts, primarily due to its anti-inflammatory, anti-cancer proliferation, apoptosis induction, anti-invasion, chemosensitization, and antioxidant effects (Choi 2019), which are predominantly linked to its relationship with OS. For instance, phloretin alleviates OS in human vascular endothelial cells (HUVECs) via the Nrf2/AMPK signaling pathway (Yang et al. 2019) and reduces mitochondrial ROS levels by activating the AMPK/Sirt3 pathway, modifying phosphorylation or acetylation of proteins in the AMPK/Sirt3/MnSOD pathway, thereby mitigating high-fat diet-induced endothelial cell OS and exerting its antioxidant effects (Han et al. 2020). However, it remains unknown whether phloretin regulates ferroptosis. Furthermore, the regulatory pathways of phloretin are diverse (Nakhate et al. 2022), yet most studies to date have employed molecular experiments or singular omics techniques to explore phloretin's functional activities, focusing predominantly on elucidating its anti-inflammatory mechanisms (Cambeiro-Pérez et al. 2021; Ren et al. 2021; Wu et al. 2019). Thus further research is needed to elucidate the molecular pathways through which phloretin regulates the process of ferroptosis using omics approaches.

Previous studies have demonstrated that phloretin can alleviate lipid peroxidation in mice and degrade the lipid peroxidation product acrolein (Liou et al. 2020; Zhu et al. 2022), suggesting that phloretin may inhibit ferroptosis. So, herein, it was hypothesized that phloretin mitigates ferroptosis by alleviating intracellular OS induced by exogenous stimuli. Therefore, both adipocytes (bovine preadipocytes and 3T3-L1) and myocytes (bovine primary myocytes and C2C12) were utilized to explore whether phloretin can inhibits RSL3-induced ferroptosis, employing transcriptomics and metagenomics to elucidate its molecular mechanisms.

## Results

### Phloretin inhibits RSL3-induced ferroptosis in mouse and bovine pre-adipocytes and myocytes

**Fig. 1:**
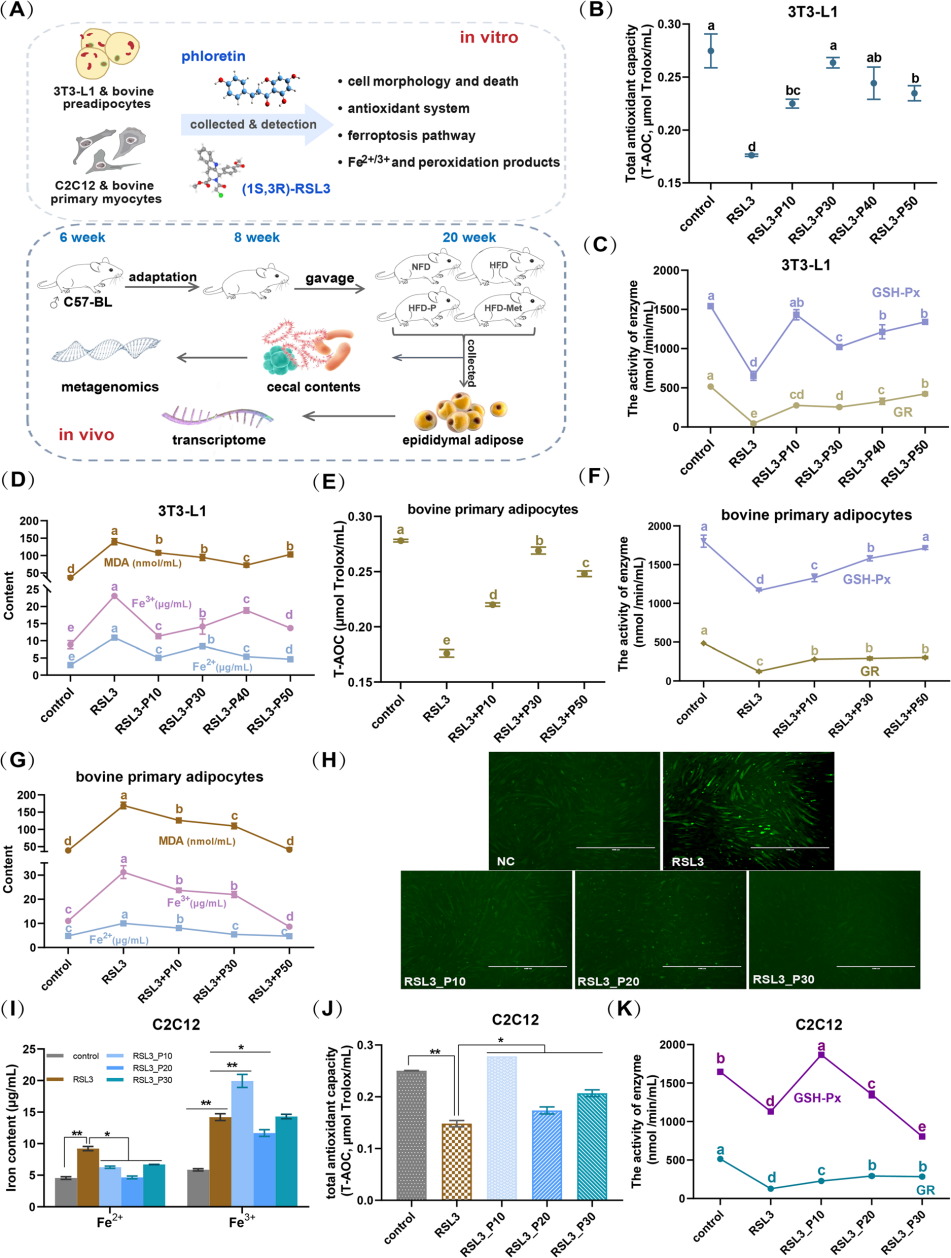
Phloretin alleviates RSL3-induced ferroptosis of primary adipocytes and myocytes. **A** The experimental design of phloretin inhibiting ferroptosis in vivo and in vitro. **B**-**G** Phloretin co-treatment significantly reversed the decline in T-AOC and GSH-PX enzyme activity, as well as the increase in lipid peroxidation product MDA and iron ion content induced by RSL3 in (**B**-**D**) mouse 3T3-L1 and (**E**–**G**) bovine primary adipocytes. **H**–**K** Phloretin also significantly reduced cellular (**H**-**I**) ROS and ferrous ion content while restoring (**J**) T-AOC and (**K**) GSH-Px and GR enzyme activity in C2C12 cells. Note: RSL3 means (1S,3R)-RSL3. * and ** indicate significant differences at *P* < 0.05 and *P* < 0.01, respectively

To evaluate the broader implications of phloretin in modulating ferroptosis, herein, multiple cellular models were utilized, comprising mouse 3T3-L1 adipocytes, C2C12 myoblasts, bovine preadipocytes and primary muscle cells (Fig. 1A). Upon treatment with the ferroptosis activator RSL3, 3T3-L1 cells exhibited decreased T-AOC (Fig. 1B), GSH-Px, and GR enzyme activities (Fig. 1C) and increased MDA, Fe^2+^, and Fe^3+^ levels (Fig. 1D), whereas co-incubation with phloretin (10–50 μmol/L) reversed these effects by elevating T-AOC, GSH-Px, and GR activities while inhibiting the rise in lipid peroxidative product levels, such as MDA, Fe^2+^, and Fe^3+^ (Fig. 1B-D). For primary bovine adipocytes, co-treatment with different concentrations of phloretin also significantly elevated intracellular T-AOC (Fig. 1E), GSH-Px, and GR enzyme activities (Fig. 1F) while inhibiting the RSL3-induced increase in MDA, Fe^2+^, and Fe^3+^ (Fig. 1G).

 inhibited the RSL3-induced elevation of ROS (Fig. 1H), Fe2+ and Fe3+ contents (Fig. 1I), and elevated T-AOC (Fig. 1J), GSH-Px and GR enzyme activities (Fig. 1K).In mouse myoblasts C2C12 cells, consistent with the findings on adipocytes mentioned above, phloretin (10–30 μmol/L) significantly inhibited the RSL3-induced elevation of ROS (Fig. 1H), Fe^2+^ and Fe^3+^ contents (Fig. 1I), and elevated T-AOC (Fig. 1J), GSH-Px and GR enzyme activities (Fig. 1K). Thus, both low and high concentrations of phloretin co-treatment effectively inhibited RSL3-induced ferroptosis in mouse and bovine pre-adipocytes and myocytes.

### Phloretin alleviates OS to mitigate exogenous stimulus-induced ferroptosis in vitro

OS is the most common inducer of ferroptosis, and phloretin has demonstrated significant antioxidant activity in HUVECs according to our reports (Han et al. 2020; Yang et al. 2019). So, it was hypothesized that phloretin mitigates intracellular OS to inhibit ferroptosis.

**Fig. 2:**
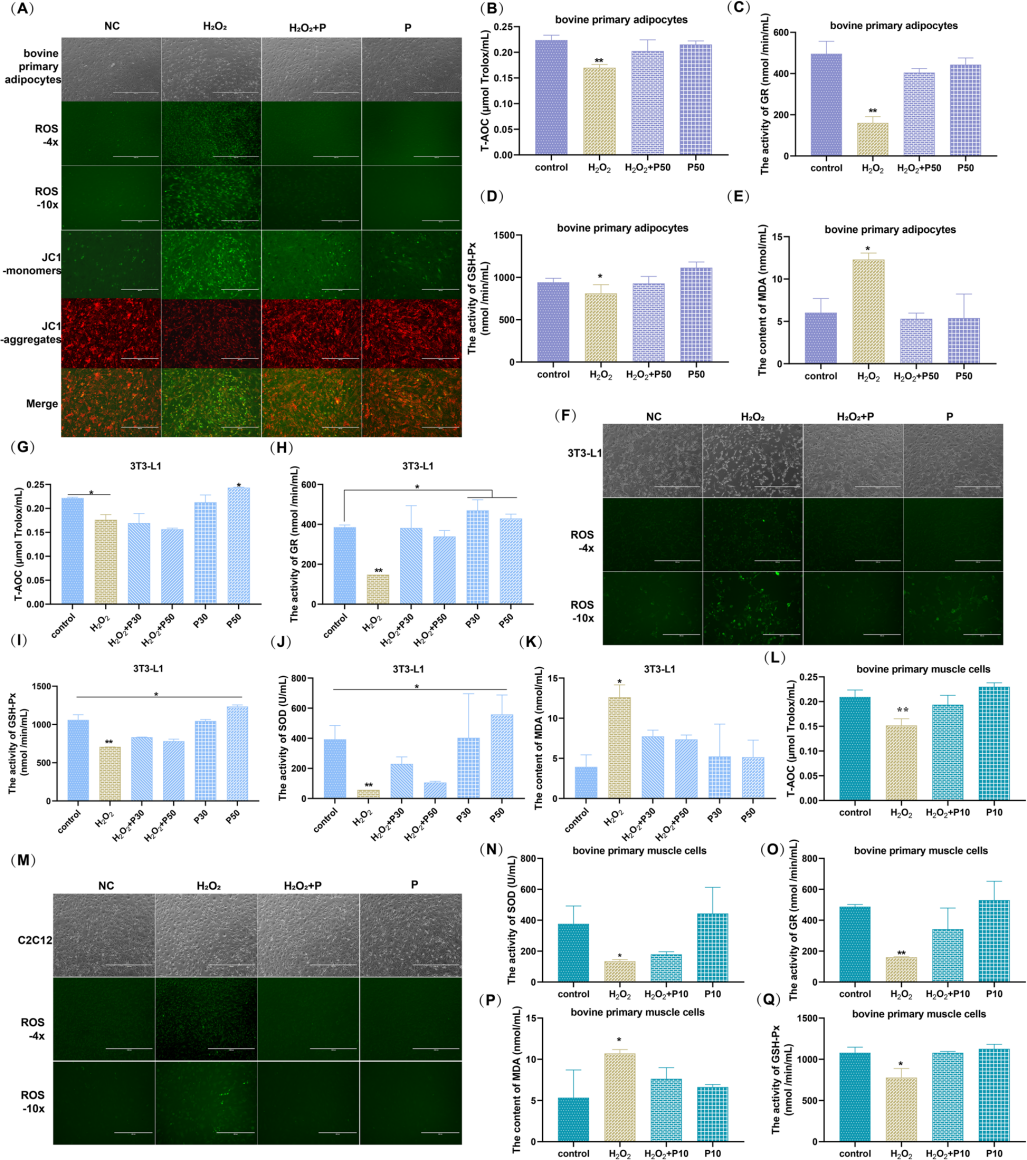
Phloretin effectively inhibits key inducers of ferroptosis, OS, in (**A**-**E**) bovine primary adipocytes, (**F**-**K**) 3T3-L1, and (**L**-**Q**) bovine primary muscle cells. **A**, **F**, **M** The change of cell morphology (4 x,1000 µm), ROS content (4 x, 1000 µm; 10 x, 400 µm), and mitochondrial membrane potential (10 x, 400 µm) in different kinds of cells with phloretin or H_2_O_2_ treatment. **B**, **G**, **L** The total antioxidant capacity, (**C**, **H**, **O**) GR enzyme (**D**, **I**, **Q**) GSH-Px enzyme, (**J**, **N**) SOD, and (**E**, **K**, **P**) MDA content of different types cells. Note: In the absence of auxiliary lines, * and ** indicate significant differences between this group and others (*P* < 0.05) or very significant difference (*P* < 0.01)

In comparison to the control, cells exhibited significant cytoplasmic contraction and a marked increase in intracellular ROS probe fluorescence intensity after external stimulation with H_2_O_2_, confirming the successful OS modeling in mouse and bovine pre-adipocytes and myocytes (Fig. 2A, F, M). But in the phloretin co-incubation and phloretin-alone groups, cell morphology resembled normal cells with significantly lower ROS levels (Fig. 2A, F, M). Additionally, OS resulted in increased JC-1 monomer green fluorescence intensity and decreased aggregate red fluorescence intensity, whereas phloretin co-treatment significantly enhanced MMP in bovine adipocytes (Fig. 2A).

**Fig. 3:**
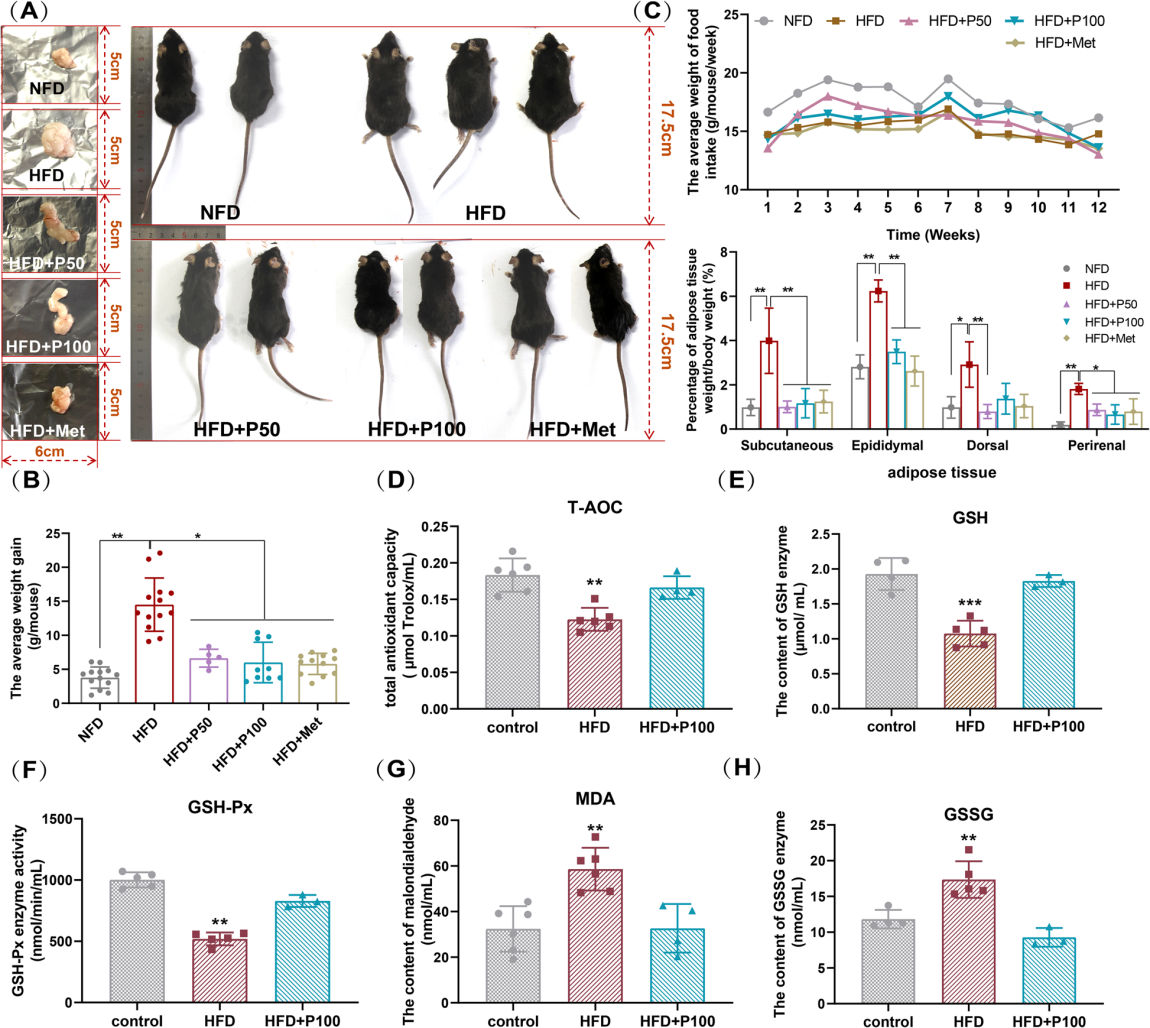
Phloretin treatment suppresses HFD-induced obesity and OS in vivo. After 12 weeks of high-fat feeding and gavage, (**A**) the morphology of mice and epididymal adipose tissue, (**B**) the average weight gain, (**C**) the trend of weekly food intake, and the weight ratio of different adipose tissues to sacrificed body weight were assessed, (**D**-**H**) alongside serum detection of redox-related parameters (collected without anticoagulant and centrifuged at low temperature). Note: In the absence of auxiliary lines, * and ** indicate significant differences between this group and others (*P* < 0.05) or very significant difference (*P* < 0.01)

Imbalance in cellular redox homeostasis, maintained by oxidases and reductases, is a direct trigger and crucial feature of OS and ferroptosis. Further assessment of key cellular redox indicators revealed that phloretin significantly improved the weakened T-AOC induced by OS, notably restored the decreased levels of GR, GSH-Px, and SOD activities, and reduced the MDA content in bovine pre-adipocytes (Fig. 2B-E) and 3T3-L1 cells (Fig. 2G-K) under H_2_O_2_-stimulation. Similarly, phloretin effectively preserved cellular morphology and rescued the activity of antioxidant enzymes and reductases in bovine primary myocytes under OS stimulation (Fig. 2L-Q), consistent with its previously demonstrated effects in mouse myoblast C2C12 (Li et al. 2020). So phloretin alleviated OS to mitigate ferroptosis in both bovine and murine preadipocytes and myoblasts, confirming its excellent antioxidant efficacy across different species and cell types.

### Phloretin alleviates HFD induced OS in the obese mice

To address disparities between in vitro cell culture conditions and the physiological environment in vivo, C57BL/6 mice were used as an obesity model through HFD feeding, with phloretin or metformin intervention administered by gavage (Fig. 1A). After 12 weeks, mice on the HFD (gavaged with 0.3% CMC-Na) exhibited higher obesity rate (Fig. 3A), greater average weight gain (Fig. 3B), and a higher body fat percentage (Fig. 3C) than those on the NFD, indicating the successful establishment of an obesity model. Notably, except for the mouse with NFD, which had a slightly higher food intake during the first 8 weeks compared to the others, there were no significant differences in the average weekly food intake among the different groups throughout the feeding and gavage period (Fig. 3C).

Furthermore, both high and low concentrations of phloretin significantly suppressed the HFD-induced increase in various fat depots, including epididymal, subcutaneous, and dorsal fat, consistent with the effects of metformin treatment (Fig. 3C), demonstrating that both low and high concentrations of phloretin effectively mitigate HFD-induced obesity in mice.

Furthermore, the T-AOC (Fig. 3D), antioxidant GSH-Px enzyme activity, and reduced GSH enzyme content of serum of obese mice were significantly lower than controls (Fig. 3E, F). Concurrently, the contents of GSSG and malondialdehyde were markedly elevated in obese mice (Fig. 3G,H), confirming the presence of OS in the obese mouse, consistent with previous reports (Tan and Norhaizan 2019). In contrast, combined with 12 weeks of phloretin gavage, phloretin effectively suppressed the HFD-induced decline in T-AOC (Fig. 3D), as well as the decrease in GSH-Px enzyme activity and GSH enzyme content of mouse serum (Fig. 3E,G), and inhibited the production of peroxidation products such as GSSG and MDA (Fig. 3G,H), revealing the effective amelioration of obesity-induced OS in mice by phloretin intervention.

### The ferroptosis of adipose and muscle tissues in obese mice is ameliorated by phloretin

The impact of obesity-induced OS on cellular ferroptosis within mice adipose and muscle tissues, and the potential modulation of these ferroptotic processes by phloretin in vivo, were further investigated.

**Fig. 4:**
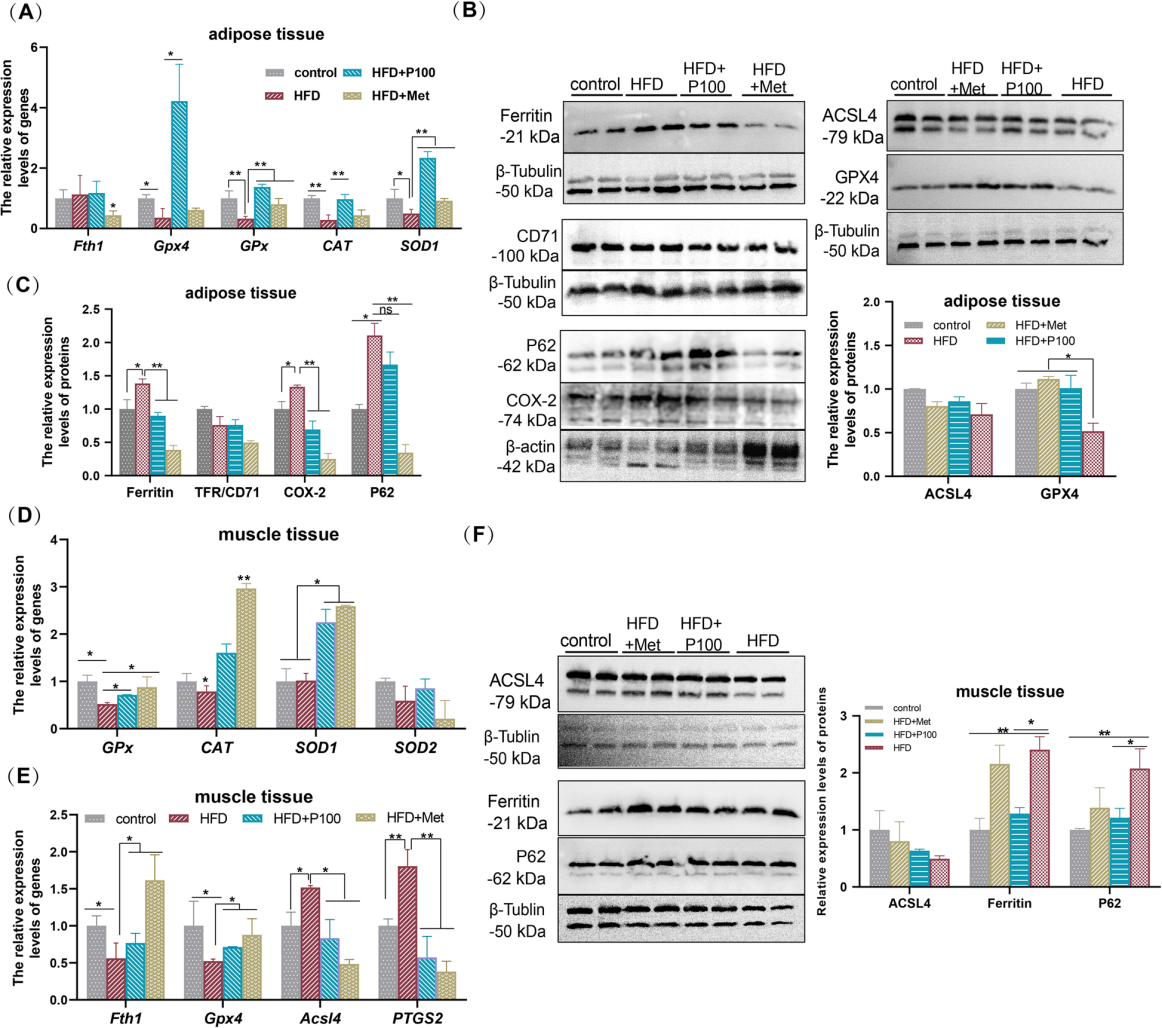
Phloretin regulates OS-induced ferroptosis in mouse epididymal adipose tissue and skeletal muscle. **A**, **D**, **E** The expression of genes encoding antioxidant enzymes or ferroptosis regulators. The expression of ferroptosis-related proteins in (**B**-**C**) adipose and (**F**) muscle tissue. Note: *, ** and *** indicate significant differences between this group and others with *P* < 0.05, *P* < 0.01 and *P* < 0.001, respectively

In the epididymal adipose tissue of obese mice, the expression levels of ferroptosis-inhibiting genes (*Fth1*, *Gpx4*) and genes encoding antioxidant enzymes such as *GPx*, *CAT*, and *SOD1* were significantly reduced, but these expressions were significantly increased in mice treated with phloretin (HFD + P100) or metformin (positive controls) (Fig. 4A). Meanwhile, protein expression analysis showed increased levels of P62 and COX2 (PTGS2) promoting ferroptosis, alongside decreased GPX4 levels in HFD mice, whereas phloretin-treated mice exhibited decreased expression of ferroptosis-promoting proteins and increased levels of ferroptosis-inhibiting proteins (Fig. 4B-C). Notably, ferritin was highly expressed in HFD mice (Fig. 4C). During severe OS, ferritin fails to effectively bind and store iron ions, leading to the release of excess iron, which catalyzes the generation of lipid peroxides and promotes ferroptosis. However, the addition of phloretin or metformin alleviates OS and inhibited the expression of ferritin (Fig. 4C).

Meanwhile, in mouse muscle tissues, phloretin significantly upregulated antioxidant enzyme-encoding genes (*GPx*, *CAT*, *SOD1*, *SOD2*) compared to HFD-induced obese mice (Fig. 4D), while also suppressing NOX1 and PTGS2 expression and attenuating the increase in Gpx4 expression associated with ferroptosis (Fig. 4E). Protein analysis in skeletal muscle showed a consistent reduction in P62 and ferritin expression in phloretin-treated mouse compared to HFD-induced obese mice (Fig. 4F), aligning with findings in mice adipose tissue. So, these indicated that obesity-induced OS triggers ferroptosis in adipose and muscle tissues, but phloretin reversed this trend.

### Phloretin exerts antioxidant activity independent of modulating murine intestinal microbiota as revealed by metagenomic sequencing analysis

**Fig. 5:**
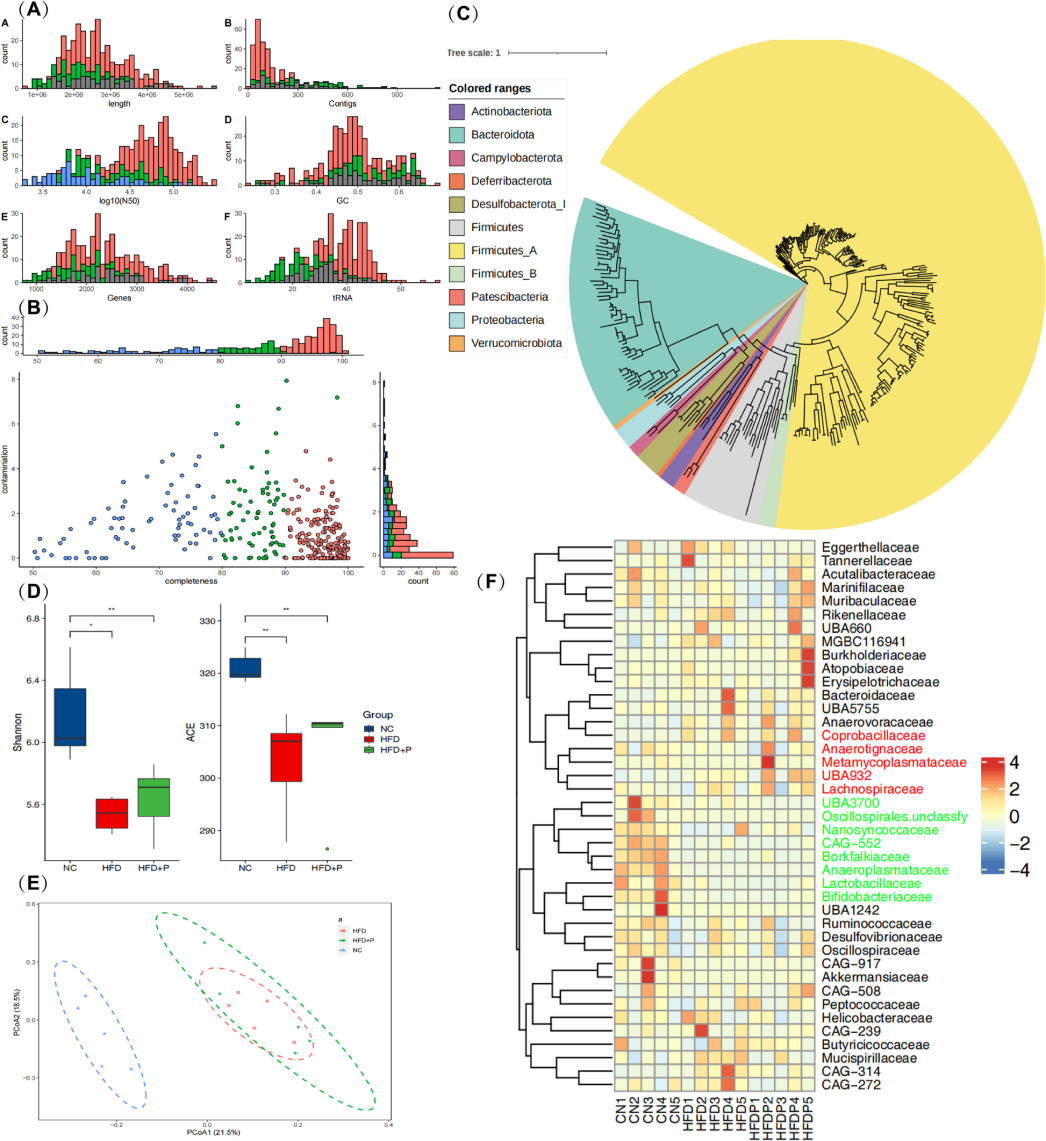
Metagenomic analysis reveals the regulation of phloretin on gut microbiota in obese mouse. **A**-**B** Assembly quality and (**C**) the phylogenetic tree of microbial genomes. The results of microbial biodiversity analysis (**D**) alpha diversity, (**E**) PCA analysis, and (**F**) clustering heat map

Considering that oxidative stress in the gut can lead to OS in other tissues or organs, cecal contents from mice were collected for metagenomic sequencing analysis. A total of 345 MAGs was recorved, including 199 nearly complete MAGs (completeness > 95%) and 73 high-quality MAGs (completeness > 80%) (Fig. 5A-B). Phylogenetic tree construction and species classification annotation of the MAGs revealed that the majority MAGs belonged to Firmicutes (244/345, Fig. 5C, yellow portion), followed by Bacteroidetes (55/345, green portion).

Further α-diversity analysis of the gut microbiota in different groups revealed a significant reduction in microbial diversity in the HFD and HFD + P groups (Fig. 5D). The PCA analysis showed that the microbial community structure in the HFD and HFD + P groups was almost identical, both differing significantly from the NC group (Fig. 5E). Compared with NC group, the relative abundances of UBA3700, Nanosyncoccaceae, and Borkfalkiaceae, were higher in the HFD or HFD + P groups, while that of Coprobacillaceae, Anaerotignaceae, Metamycoplasmataceae, UBA932, and Lachnospiraceae were higher in the HFD or HFD + P groups (Fig. 5F). Accordingly, high-fat diet feeding markedly altered the diversity and structure of gut microbiota, while phloretin supplementation does not alleviate these changes, suggesting that the antioxidant activity of phloretin was independent of modulating murine intestinal microbiota.

### Transcriptomic analysis reveals phloretin alleviates ferroptosis of adipocyte tissue by AMPK-PPAR pathways

**Fig. 6:**
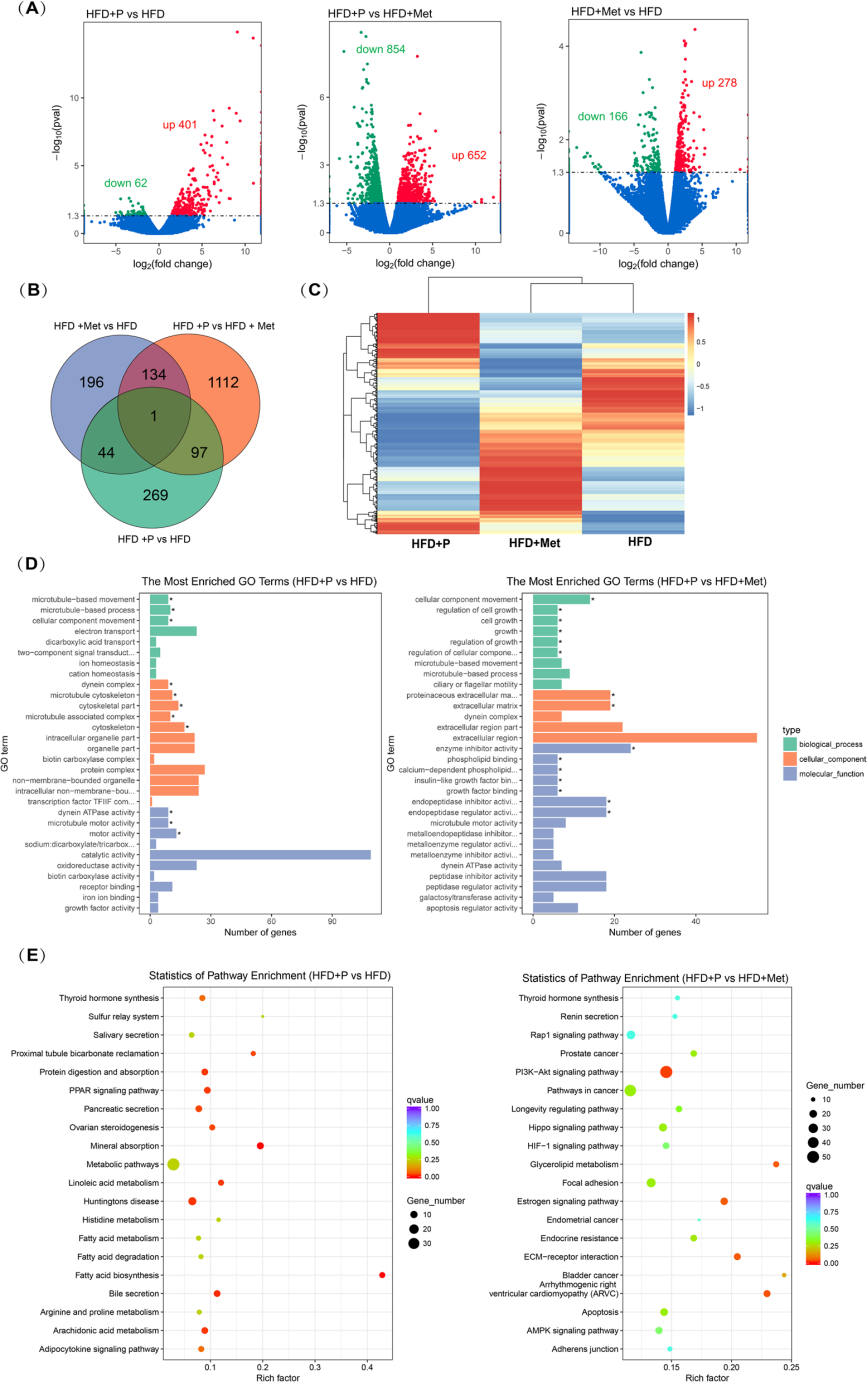
Sequencing analysis of differentially expressed mRNA (de-mRNA) in epididymal adipose tissue with HFD or phloretin co-treatment. **A** The volcano map and (**B**) venn map of differentially expressed genes (de-genes). **C** The clustering heat map of different groups. **D** The GO enrichment analysis and (**E**) KEGG enrichment analysis of de-mRNAs in HFD + P vs HFD or HFD + P vs HFD + Met

Whole transcriptome sequencing identified 411 differentially expressed genes (de-genes) between the HFD + P and HFD groups (Fig. 1A-B), with 352 genes upregulated and 59 genes downregulated (Fig. 6 A-B), including *Gpx3*, adenylate kinase 7 (*AK7*) and 9 (*AK9*), *Slc40a1* (iron-regulated transporter), *FABP7* (fatty acid binding protein 7), *Slc27a1* (fatty acid transporter) and other solute carrier family genes (Table S2-S3). Compared with the positive control treated with metformin, phloretin significantly reduced the expression of proliferation-related genes (e.g., *Cdkn1a*, *PCNA*), NADPH oxidase 4 (*NOX4*), leptin, and lipase (Table S4). Meanwhile, the expression levels of energy metabolism-related genes such as ATPase type 11 A (*Atp11a*), ATP/GTP binding protein-like 5 (*Agbl5*), adenylate kinase 9 (*AK9*), and calcium/calmodulin-dependent protein kinase kinase 2 (*Camkk2*) were significantly higher in the phloretin co-treatment mouse than in the metformin gavage mouse (Table S5). Notably, as an upstream regulator of AMPK, the central modulator of cellular energy, CAMKK2 is activated by phloretin, leading to the activation of the AMPK/Nrf2 signaling pathway to alleviate cellular oxidative stress, a mechanism that has been repeatedly demonstrated in our previous work (Han et al. 2020, 2021; Li et al. 2020; Yang et al. 2019).

Hierarchical clustering and PCA indicated pronounced expression differences in HFD + P_vs_HFD compared to HFD + Met_vs_HFD, highlighting that phloretin (recovery group) exerts broader regulatory effects compared to metformin (positive control), potentially influencing distinct biological pathways (Fig. 6C). Further GO enrichment analysis revealed that the de-genes of HFD + P_vs_HFD were enriched in biological processes such as electron transport and ion homeostasis, as well as molecular functions related to maintaining redox homeostasis and iron ion homeostasis, including dynein ATPase activity, catalytic activity, oxidoreductase activity, and iron ion binding (Fig. 6D, Fig. 1C).

Additionally, KEGG enrichment analysis of HFD + P_vs_HFD demonstrated significant enrichment of de-genes in pathways related to lipid synthesis, fatty acid biosynthesis, metabolism, and degradation. Moreover, those de-genes were also enriched in the AMPK signaling pathway, and PPAR signaling pathway in HFD + P_vs_HFD group (Fig. 6E). Notably, P13K-Akt, apoptosis and AMPK signaling pathway were enriched pathway of HFD + P_vs_HFD + Met (Fig. 6E, Fig. 1D). Furthermore, the activation of the AMPK signaling pathway by phloretin to alleviate cellular oxidative stress has been consistently demonstrated in our prior studies (Han et al. 2020, 2021; Li et al. 2020; Yang et al. 2019). Therefore, based on the transcriptomic analysis and previous findings, it was supported that phloretin alleviates OS through the AMPK-PPAR pathway, thereby inhibiting ferroptosis in adipocytes and myocytes.

## Discussion

Unlike reports which suggest that bioactive substances (e.g., phloridzin) exert anti-inflammatory and antioxidant effects by altering gut microbiota and their metabolites (Cheng et al. 2023; Wu et al. 2019; Zhang et al. 2020), this study found that phloretin alleviated HFD-induced reduction in gut microbial diversity in mice but did not restore it to normal levels, and had no significant impact on the gut microbiota structure in HFD mice, suggesting that phloretin's antioxidant effects are primarily mediated by inducing changes in gene expression and protein activity within cells rather than by modulating gut microbiota. As a small molecule compound, phloretin has been proven to bind directly to proteins and other molecules to exert its biological activity (Chen et al. 2020; Ying et al. 2019). For instance, phloretin directly insert into the active center of tyrosinase, forming four hydrogen bonds with surrounding amino acid residues, thereby occupying the active site, altering the secondary structure of tyrosinase, and consequently reducing its catalytic activity (Zhang et al. 2018). Herein, although it remains unconfirmed whether phloretin directly targets these processes, phloretin significantly inhibited oxidase activity and altered the transcription of genes enriched in AMPK-PPAR pathway, such as *Camkk2*. In previous studies, phloretin promotes AMPK phosphorylation at Thr172, enhancing the expression or activity of downstream p-Nrf2/HO-1 or Sirt3 proteins, then reducing lysine acetylation of MnSOD, and restoring its enzymatic activity, thereby inhibiting cellular OS (Han et al. 2020; Yang et al. 2019). Moreover, phloretin can improve insulin sensitivity by blocking the Ser273 site of PPARγ and inhibiting the auto-activation of CDK5, disrupting the PPARγ/CDK5 interaction in differentiated adipocytes (Shiv et al. 2019). Therefore, transcriptomic analyses in this study revealed that phloretin inhibits cellular ferroptosis by restoring redox homeostasis and further suppressing OS, rather than altering ion concentrations or gut microbiota, through the regulation of protein modifications and key signaling pathways. Additionally, this study sampled cecal contents, which have the most microbial abundance, for metagenomics analysis, whereas previous studies sampled feces or colonic contents (Chen et al. 2020; Cheng et al. 2023), potentially accounting for differences in microbiota profiles.

Given the complexity of living organisms, single-omics analysis is scarcely possible to fully reveal the fundamental principles of biological processes. Currently, omics-based research on the mechanisms of phloretin is limited and mostly focuses on its anti-inflammatory effects (Cambeiro-Pérez et al. 2021; Ren et al. 2021). Herein, transcriptomics and metagenomics were utilized to identify the primary signal pathways through which phloretin exerts its antioxidant activity, such as AMPK-PPAR signaling pathways, which also regulate adipogenesis, cellular energy metabolism, and ferroptosis-related pathways. It has been reported that under cellular stress, AMPK activation leads to the phosphorylation and inactivation of acetyl-CoA carboxylase, thereby limiting the biosynthesis of PUFAs and other fatty acids, ultimately inhibiting ferroptosis (Lee et al. 2020; Li et al. 2025). Transcriptome in this study supported that phloretin activated AMPK-PPAR signaling pathways to reduce OS-induced ferroptosis. Furthermore, saponins, terpenoids, and alkaloids induce ROS and ferritinophagy-dependent ferroptosis, while flavonoids and polyphenols inhibit ferroptosis by modulating iron metabolism and Nrf2 signaling (Zheng et al. 2021). As a dihydrochalcone compound, phloretin likely mitigates RSL3-induced ferroptosis by regulating Nrf2 activity. Our previous research demonstrated that phloretin promotes the dissociation of the Keap1/Nrf2 complex, facilitating Nrf2 nuclear translocation (Li et al. 2020), consistent with reports that phloretin prevents cardiomyopathy by inhibiting oxidative stress through Keap1/Nrf2 complex dissociation (Ying et al. 2018). AMPK downstream targets include key antioxidant regulators such as GPX4 and PPARγ (Chen and Maltagliati 2018), which is also regulated by AMPK (Chyau et al. 2020; Lee et al. 2023; Yang et al. 2023). Our transcriptomic analysis also propound AMPK and PPARγ as targets regulated by phloretin, but their direct or indirect interactions remain to be elucidated.

Beyond its aforementioned antioxidative stress properties, phloretin was demonstrated for the first time in this study to effectively inhibit ferroptosis in both tissues and cells. As a key inhibitor of ferroptosis, GPX4 reduces toxic lipid hydroperoxides (PLOOH) to non-toxic lipid alcohols (Bersuker et al. 2019). Herein, phloretin treatment significantly enhanced both transcriptional and translational activity of GPX4 in HFD-fed mice. The increased GPX4 activity further suppressed ACSL4 transcription via negative feedback (Doll et al. 2017), which was verified in skeletal muscle tissue of phloretin-gavaged mice in this study, thereby inhibiting the generation of pro-ferroptotic lipid substrates (e.g. PUFA-PLs) and reducing cellular susceptibility to ferroptosis (Doll et al. 2017). Concurrently, phloretin-mediated inhibition of ferroptosis attenuated lipid peroxide-activated inflammatory pathways, leading to downregulated COX-2 expression (Luo et al. 2022). However, this study is limited to detecting changes solely at the transcriptional and protein expression levels of ferroptosis pathway proteins. Whether phloretin directly binds to or indirectly regulates key ferroptosis protein such as GPX4 requires further investigation.

As an easily absorbed small molecule dihydrochalcone compound, phloretin exhibits low toxicity, anti-inflammatory, antioxidant, and ferroptosis-inhibitory activities, indicating its broad application potential (Mariadoss et al. 2019). Its anti-ferroptosis properties suggest it could mitigate neural cell damage and death, thereby slowing the progression of neurodegenerative diseases, and potentially enhancing cancer cell sensitivity to improve chemotherapy and radiotherapy outcomes. Furthermore, oxidative stress-induced dysfunction of the oxidative stress sensor NPGPx in adipocytes leads to obesity in mice and humans (Chang et al. 2013). Phloretin treatment can regulate serum levels of leptin, adiponectin, triglycerides, LDL, and free fatty acids in obese mice (Liou et al. 2020), aligning with our findings and suggesting its potential in treating obesity and metabolic disorders. In livestock, adding phloretin to feed or administering it through injections could alleviate stress-induced by inflammatory, extreme temperatures (e.g., heat stress), and long-distance transport, thereby preventing meat quality deterioration, lipid peroxidation, and immune suppression. However, long-term effects and safety of phloretin clinical or production application require further evaluation. Despite our findings on phloretin's inhibition of ferroptosis, the interactions between target molecules identified through multi-omics analyses remain unvalidated experimentally. Future research should further explore these interactions to optimize phloretin's mechanisms and molecular networks.

## Conclusion

In summary, based on multi-omics analysis combined with in vivo and in vitro verification, phloretin effectively alleviate the OS and further inhibiting ferroptosis of adipose or muscle cells in bovine and mice through AMPK-PPAR pathway, which can provide new research ideas for ameliorating adipose or myocyte dysfunction induced by ferroptosis.

## Materials and methods

### Preparation of reagents for in vitro and in vivo treatments

In vitro, the solid analytical pure forms of phloretin (HPLC ≥ 98%, B20450, Yuanye) and (1S,3R)-RSL3 (S81241, Yuanye) were dissolved in freshly opened dimethyl sulfoxide (DMSO) and subsequently diluted with serum-free medium or D-PBS before being added to the cell culture dishes. A 30% hydrogen peroxide solution was filtered through a 40 μm cell strainer and then diluted with sterile PBS resulting in a final concentration of 500 μmol/L for the cell experiments. For the gavage administration in mice, phloretin (P127748, Aladdin) or metformin hydrochloride (M107827, Aladdin) was dissolved in the 0.3% carboxymethyl cellulose sodium (CMC-Na, C104986, Aladdin) solution.

### Isolation and culture of primary adipocytes and muscle cells

Fetal bovine skeletal muscle and subcutaneous adipose tissues from the abdomen or inguinal region were repeatedly washed with fresh PBS and minced (Jiang et al. 2025). The skeletal muscle and subcutaneous adipose tissues were digested with 0.2% type II and I collagenase for 1 to 1.5 h, respectively. Then they were centrifuged, and the supernatant was filtered through the 40 μm cell strainer. After several washes and centrifugations with serum-free DMEM, the precipitate was resuspended in culture medium with 20% FBS and transferred to culture dishes. Myocytes were purified using differential adhesion: non-adherent cells were transferred after 2 h of culture, repeated 2–3 times.

The isolated primary bovine adipocytes were cultured with medium consisting of 20% FBS, 79% high-glucose medium (H-DMEM)/F12 medium and 1% penicillin–streptomycin, while the medium for primary bovine myocytes was slightly different, containing 10% FBS, 89% H-DMEM medium and 1% penicillin–streptomycin, under 37 °C with 5% CO_2_. Moreover, the mouse preadipocyte cells (3T3-L1, SCSP-5038), provided from the Cell Bank of the Chinese Academy of Sciences (Shanghai, China), were cultured in a medium consisting of 10% calf serum (CS), 89% H-DMEM, and 1% penicillin–streptomycin at 37 °C and 7% CO_2_. In contrast, the mouse myoblast cells (C2C12, also known as embryonic myoblast, SCSP-505) were cultured in the medium containing 10% FBS, 89% H-DMEM, and 1% penicillin–streptomycin at 37 °C and 5% CO_2_, respectively.

### Mouse feeding and experimental treatment

Nearly 60 male C57BL/6 J mice (6 weeks old, from the Experimental Animal Center of Xi'an Jiaotong University) were adaptively fed for 2 weeks under controlled conditions (22 ± 2 °C, 55 ± 5% humidity, 12-h light/dark cycle) and then randomly divided into five groups: normal diet (NFD), high-fat diet (HFD), HFD with low-dose phloretin gavage (50 mg/kg, HFD + P50) (Dierckx et al. 2022), HFD with high-concentration phloretin gavage (100 mg/kg, HFD + P100) (Han et al. 2021), and HFD with metformin (200 mg/kg, HFD + Met, positive control) (Zeng et al. 2019).

The NFD group received a standard diet (4% fat, 12.5% protein, 72.7% carbohydrates; AIN93G, Trophic Animal Feed High-tech Co. Ltd), while the HFD mouse received a diet (60% fat calories, 14.1% protein calories, 25.9% carbohydrate calories; TP23400, Trophic Animal Feed High-tech Co. Ltd) to induce obesity. All mice had free access to food and water, and food intake and body weight were recorded daily. After 12 weeks of gavage, mice were sacrificed after anesthesia, and blood, adipose tissues, skeletal muscle and cecal contents were collected (Fig. 1A).

### Detection of redox enzyme activities and lipid peroxidation products contents

Moreover, serum samples were analyzed for redox-related parameters using the corresponding kit, including the total antioxidant capacity (T-AOC), the content of reduced glutathione (GSH), oxidized glutathione disulfide (GSSG) and malondialdehyde (MDA), and the activity of glutathione reductase (GR) and glutathione peroxidase (GSH-Px).

The change of redox-related enzyme activity and lipid peroxidation products in cells or serum were measured using assay kits from Beyotime Biotechnology (China), including the total antioxidant capacity assay kit with ABTS (S0119), glutathione reductase assay kit with DTNB (S0055), GSH and GSSG assay kit (S0053), cellular glutathione peroxidase assay kit with DTNB (S0057S), total superoxide dismutase (SOD) assay kit with WST-8 (S0101), ROS (S0033M) and MDA (S0131M) assay kit, respectively.

### Ion content detection in cells

Trivalent iron dissociates from complexes in acidic conditions and is then reduced to divalent iron using a reducing agent. Divalent iron ions react with ferrozine to form a purple-colored compound, which shows a characteristic absorption peak at 562 nm, facilitating the quantification of iron (Fe^3+^) or ferrous ion (Fe^2+^) content. Cells subjected to different treatments were collected, centrifuged to remove supernatant, and approximately 5 million cells were added to 1 mL of extraction solution. Cells were disrupted using ultrasound (ice bath, 200 W power, 3 s ultrasound followed by 10 s intervals, repeated 30 times); then centrifuged at 12,000 rpm at 4 °C for 10 min, with the supernatant collected, placed on ice, and analyzed using a spectrophotometer (preheated for 30 min, wavelength set to 562 nm).

### Western blot and qRT-PCR

The collected epididymal adipose and muscle tissues from mice were immersed in liquid nitrogen and then homogenized. RNA and protein were extracted using the phenol–chloroform method and RIPA-PMSF buffer (R0010, Solarbio, China), respectively. Subsequently, RNA was reverse transcribed into cDNA using the PrimeScript™ II High Fidelity RT-PCR Kit (R023A, Takara). For protein, the samples were denatured by boiling in SDS-PAGE loading buffer (with DTT, P1015, Solarbio) and stored at −80 °C.

The qRT-PCR primer sequences, designed using the online PrimerQuest™ Tool and synthesized by Sangon Biotech (Shanghai), were validated for specificity through PCR and gel electrophoresis, with only those confirmed to be specific used in subsequent experiments, as detailed in Supplementary Table 1. PCR was performed using TB Green® Fast qPCR Mix (RR430A, Takara) and a real-time quantitative PCR instrument (CFX Opus 96, Bio-Rad).

SDS-PAGE gels were prepared according to the protocol of Omni-Easy™ One-step Color PAGE Gel Rapid Preparation Kit (PG212, Epizyme). The used specific antibodies, included anti-ACSL4/FACL4 (TD12141, Abmart), anti-Ferritin (T55648, Abmart), anti-Gpx4 (PTM-6775, PTM BIO), anti-β-Tubulin (M20005, Abmart), anti-CD71/TFR1 (PTM-6294, PTM BIO), anti-Cox2 (TA7003, Abmart), anti-SQSTM1/p62 (PTM-5483, PTM BIO), and anti-β-actin (PTM-5028, PTM BIO), were diluted in universal antibody dilution buffer (PS119, Epizyme) at a ratio of 1:500 to 1:1000, while goat anti-mouse/rabbit IgG(H + L) (HRP conjugated, LF101/LF102, Epizyme) was diluted at 1:2000.

### Metagenomic Sequencing and Genome Catalog Constructing

Cecal contents from control (NC), HFD-obese mice, and HFD + P100 mice were collected and stored in liquid nitrogen (Zhou et al. 2024). A total of 15 cecal content samples, five biological replicates per group, were subjected to microbial DNA extraction (Yu and Morrison 2004), and then sequenced using the MGI DNBSEQ-T7 platform (MGI Tech Co., Ltd., Shenzhen, China) with 15 Gb per sample.

The metagenomic data were cleaned using fastp (v0.23.4) (Chen et al. 2018), and then assembled (via megahit), binned (–metabat2, –maxbin2, and –concoct options), and bin-refined (-c 50—× 10) using the metaWRAP (v1.3.2) pipeline (Uritskiy et al. 2018). The metagenomes within each block were co-assembled and co-binned. The acquired metagenome-assembled genome (MAG) were retained based on CheckM (v1.2.2) quality assessment, meeting the genome quality criteria: quality score (completeness – 5 × contamination + log(N50)) ≥ 50, completeness > 50%, and contamination < 10% (Parks et al. 2015). The retained MAG were dereplicated twice at < 99% average nucleotide identity to generate a strain-level MAG catalog using dRep (v3.4.3) with the options –S_algorithm ANImf -sa 0.99 -nc 0.2 -comW 1 -conW 5 -N50W 1 (Olm et al. 2017). Taxonomic annotation of the MAG was performed using GTDB-Tk (v1.7.0) based on the v205 database (Parks et al. 2018). The concatenated marker genes form GTDB-Tk was used for maximum-likelihood phylogenetic tree construction by iq-tree (v2.1.4-beta) (Minh et al. 2020).

### Microbial Diversity and Community Analysis

The relative abundance of MAG was calculated from standardized read coverage in each sample using the Quant_bins module of metaWRAP, and expressed as copies per million (Uritskiy et al. 2018), similar to transcripts per million in RNAseq.

The alpha diversity of MAG was calculated using the abundance-based coverage estimator (ACE) and shannon diversity index with the vegan package in R (v.4.3.2). The beta diversity of microorganisms was assessed through principal coordinate analysis plots based on Bray–Curtis dissimilarity (Bray and Curtis 1957) in R. Multivariate analysis of variance was performed using the permutational multivariate analysis of variance function from the vegan package in R to evaluate statistical differences in microbial structure across treatments. Differential analysis of microbial abundance was performed using edgeR with the glmQLFit functions (Chen et al. 2025), and the *P*-values were adjusted using the Benjamini & Hochberg method (Kovacs et al. 2011).

### Transcriptome sequencing and annotation

Mouse epididymal adipose tissues from the HFD, HFD + P100, and HFD + Met groups were collected and grounded with liquid nitrogen. Allwegene Tech. (Beijing, China) was commissioned to total RNA extract, complete cDNA library construction and high-throughput sequencing by Illumina NovaSeq 6000. After the connector sequence and low-quality reads (rate of N base > 10%, quality score < 20) were removed, HISAT2 (v2.1.0) software (https://daehwankimlab.github.io/hisat2/) was used to convert the original reads into clean reads aligned with the mouse reference genome (GRCm38) to obtain mapping data. The obtained clean reads were assembled using StringTie (v2.1.1) (Kovaka et al. 2019). The FPKM method was used to calculate the expression of each annotated gene. Differential expression analysis was performed with the DESeq2 (Love et al. 2014). Differentially expressed genes (DEGs) were screened by P-value < 0.05 and |log2(FoldChange)|≥ 1. Finally, Gene Ontology (GO) and Kyoto Encyclopedia of Genes and Genomes (KEGG) analysis were conducted by the R package clusterProfiler 3.14.3 to identify critical pathways (Kanehisa et al. 2023; Sun et al. 2023; Yu et al. 2012). The adjust *P*-values < 0.05 denoted statistical significance.

## Supplementary Information

Supplementary Material 1: Supplemental Fig. 1. The supplemental results of sequencing analysis of de-mRNAs. (A) The abundence of de-genes in HFD, HFD + P and HFD + Met groups; (B) PCA analysis; (C) The KEGG enrichment analysis and (D) GO enrichment analysis of de-mRNAs in HFD + Met_vs_HFD.

Supplementary Material 2: Supplemental Table 1. Primer sequences of mouse ferroptosis genes for qRT-PCR. Supplemental Table 2. The de-genes with downregulated expression of HFD + P_vs_HFD. Supplemental Table 3. The de-genes with up-regulated expression of HFD + P_vs_HFD. Supplemental Table 4. The de-genes with down-regulated expression of HFD + P_vs_HFD + Met. Supplemental Table 5. The de-genes with up-regulated expression of HFD + P_vs_HFD + Met. Supplemental Table 6. The de-genes with down-regulated expression of HFD + Met_vs_HFD. Supplemental Table 7. The de-genes with up-regulated expression of HFD + Met_vs_HFD

## Data Availability

Supporting Information is available from the supplemental materials or from the cor-responding author upon reasonable request.

## Abbreviations

3T3-L1: Mouse pre-adipocyte cell
AMPK: AMP-activated protein kinase
T-AOC: Total antioxidant capacity
C2C12: Mouse myoblasts cell
DMEM: High-glucose medium
DMSO: Dimethyl sulfoxide
GO: Gene ontology
GR: Glutathione reductase
GSH: Reduced glutathione
GSH-Px: Glutathione peroxidase
GSSG: Oxidized glutathione disulfide
HDL-C: High-density lipoproteincholesterol
HFD: High-fat diet
HO-1: Heme oxygenase-1
HUVECs: Human vascular endothelial cells
LDL-C: Low-density lipoprotein cholesterol
MDA: Malondialdehyde
NFD: Normal diet
NOX1: NADPH oxidase 1
OS: Oxidative stress
P/Ph: Phloretin
PTGS2/COX-2: Prostaglandin-endoperoxide synthase 2
PUFAs: Polyunsaturated fatty acids
ROS: Reactive oxygen species
RSL3: (1S,3R)-RSL3
SOD: Superoxide dismutase
TCHO: Total cholesterol
TFR/CD71: Transferrin receptor
TG: Triglyceride
