# Supplementary figures and images for: Phloretin inhibits ferroptosis by restoring the antioxidant capacity of bovine adipose and muscle cells via the AMPK-PPAR signaling pathway
Source: Stress Biol. 2025 Dec 8;5(1):74. doi: 10.1007/s44154-025-00263-4 (PMC12682731; doi:10.1007/s44154-025-00263-4)

**Supplemental Fig. 1**

**
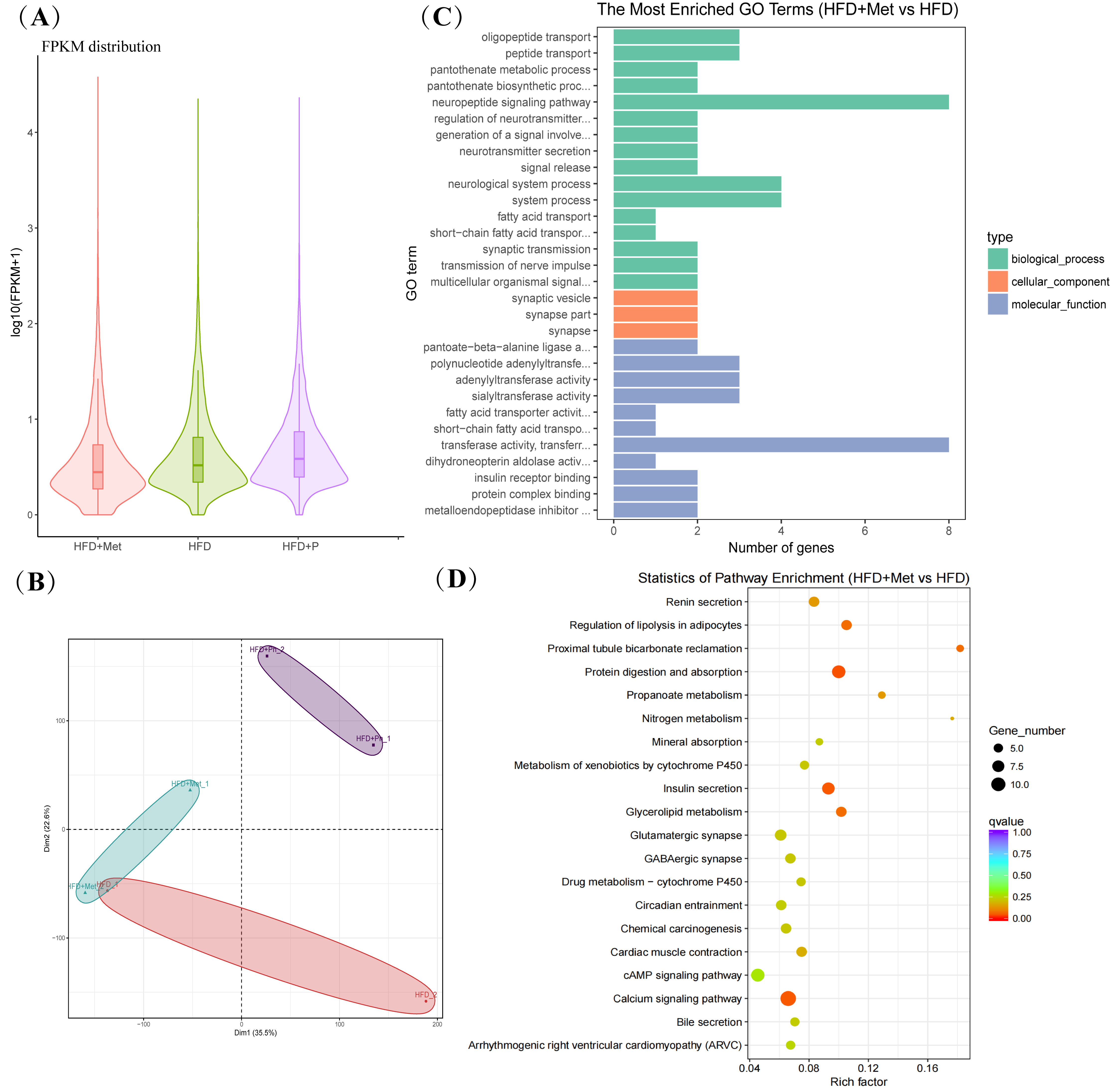
**

Supplement: Supplementary file 1 — Supplementary Material 1: Supplemental Fig. 1. The supplemental results of sequencing analysis of de-mRNAs. (A) The abundence of de-genes in HFD, HFD + P and HFD + Met groups; (B) PCA analysis; (C) The KEGG enrichment analysis and (D) GO enrichment analysis of de-mRNAs in HFD + Met_vs_HFD. [file 44154_2025_263_MOESM1_ESM.doc]
